# Correction: An atypical F-type ATPase is necessary for the function of the antibody cleavage system MIB-MIP in mycoplasmas

**DOI:** 10.1371/journal.ppat.1014203

**Published:** 2026-05-11

**Authors:** The PLOS Pathogens Editors

The originally published Acknowledgements section of [1] is incomplete. The correct Acknowledgements section is as follows:

First author JB could not be reached to confirm approval of this article for publication. Corresponding author, Yonathan Arfi (YA), affirms that JB meets the journal’s criteria for authorship, that JB’s contributions are accurately reported, and that they are not aware of any undisclosed Competing Interests for JB. YA agrees to be accountable for the article in its entirety, including the contributions of JB. The authors would like to thank Dr. Florence Tardy for kindly providing the infected goat serum. The corresponding author would like to thank Leeroy Dusi-Lance for fruitful discussions on the F1-like X0 ATPase. This study was funded by the French National Agency for Research (ANR) grant ANR-21-CE44–0002 ENIgMA.

The publisher apologizes for the error.

## References

[1] Berlureau J, Anger R, Beaulieu E, Gourgues G, Bataille L, Jaubert J, et al. An atypical F-type ATPase is necessary for the function of the antibody cleavage system MIB-MIP in mycoplasmas. PLoS Pathog. 2026;22(3):e1014043. doi: 10.1371/journal.ppat.1014043 41811866 PMC12994834

