# Supplementary figures and images for: An atypical F-type ATPase is necessary for the function of the antibody cleavage system MIB-MIP in mycoplasmas
Source: PLoS Pathog. 2026 Mar 11;22(3):e1014043. doi: 10.1371/journal.ppat.1014043 (PMC12994834; doi:10.1371/journal.ppat.1014043)

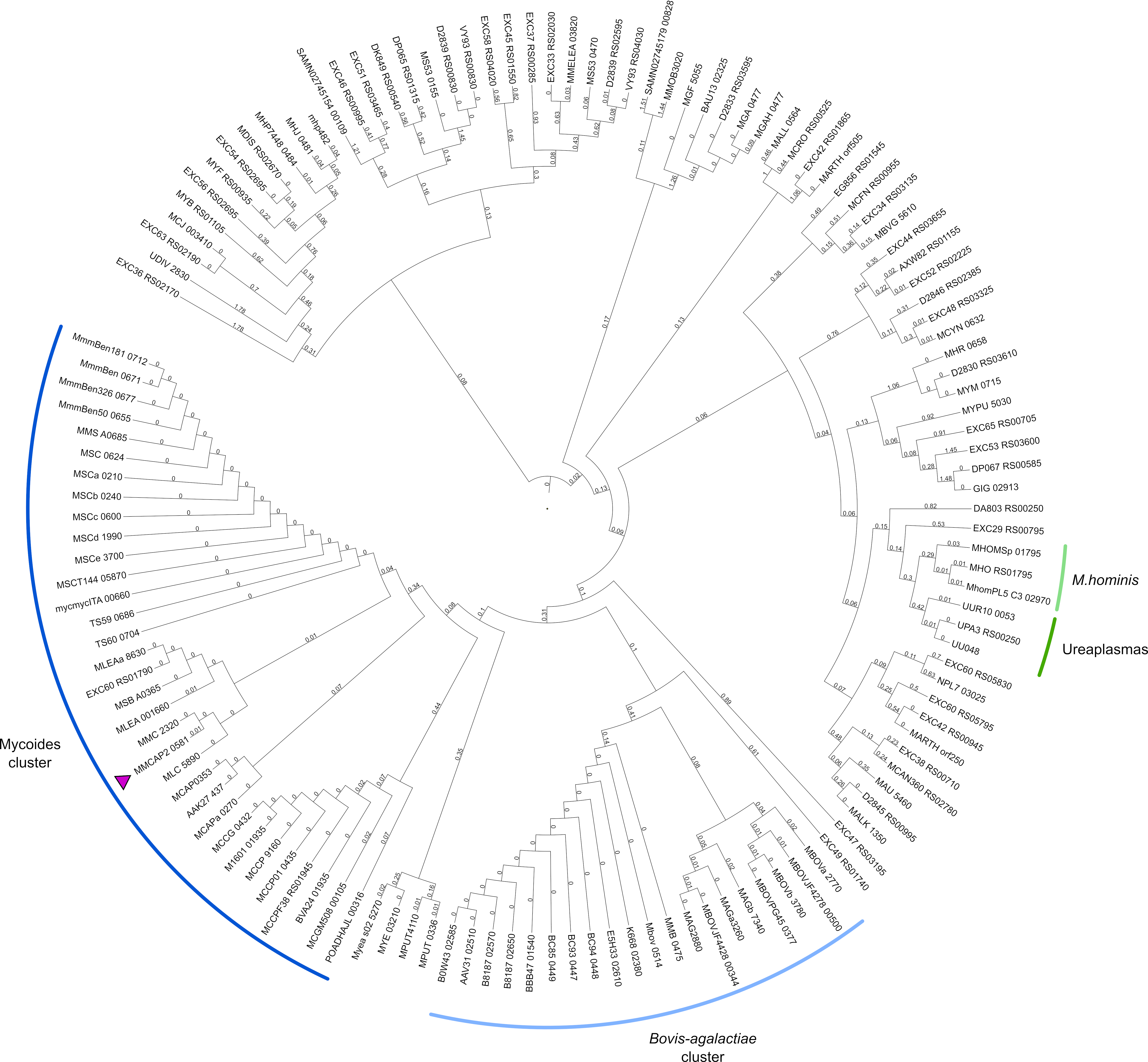

Supplement: S2 Fig — Phylogenetic analysis of the predicted homologs of MMCAP2_0581 was performed using the Phylogeny tool (https://www.phylogeny.fr/) in “One click” mode. The output phylogenic tree was visualized using iTol (https://itol.embl.de/) as a circular tree. The position of MMCAP2_0581 in the tree is marked by the coloured arrowhead. Mycoides cluster and the Bovis-agalactiae cluster are highlighted in blue, showing their phylogenetic proximity, in accordance with a horizontal gene transfer scenario, and similarly for the Hominis and Ureaplasma clusters that are highlighted in green. (TIF) [file ppat.1014043.s002.tif]

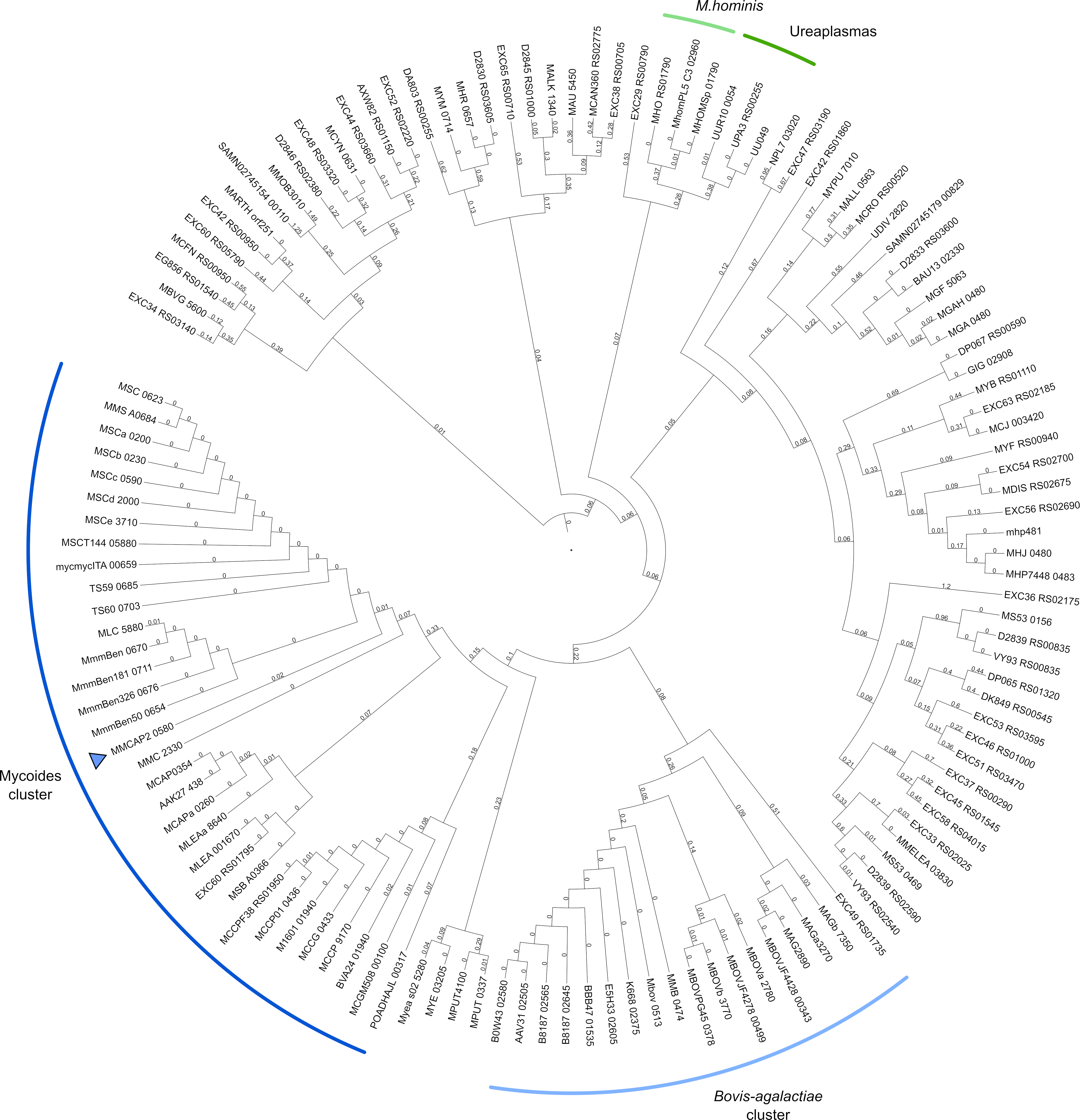

Supplement: S3 Fig — Phylogenetic analysis of the predicted homologs of MMCAP2_0580 was performed using the Phylogeny tool (https://www.phylogeny.fr/) in “One click” mode. The output phylogenic tree was visualized using iTol (https://itol.embl.de/) as a circular tree. The position of MMCAP2_0580 in the tree is marked by the coloured arrowhead. Mycoides cluster and the Bovis-agalactiae cluster are highlighted in blue, showing their phylogenetic proximity, in accordance with a horizontal gene transfer scenario, and similarly for the Hominis and Ureaplasma clusters that are highlighted in green. (TIF) [file ppat.1014043.s003.tif]

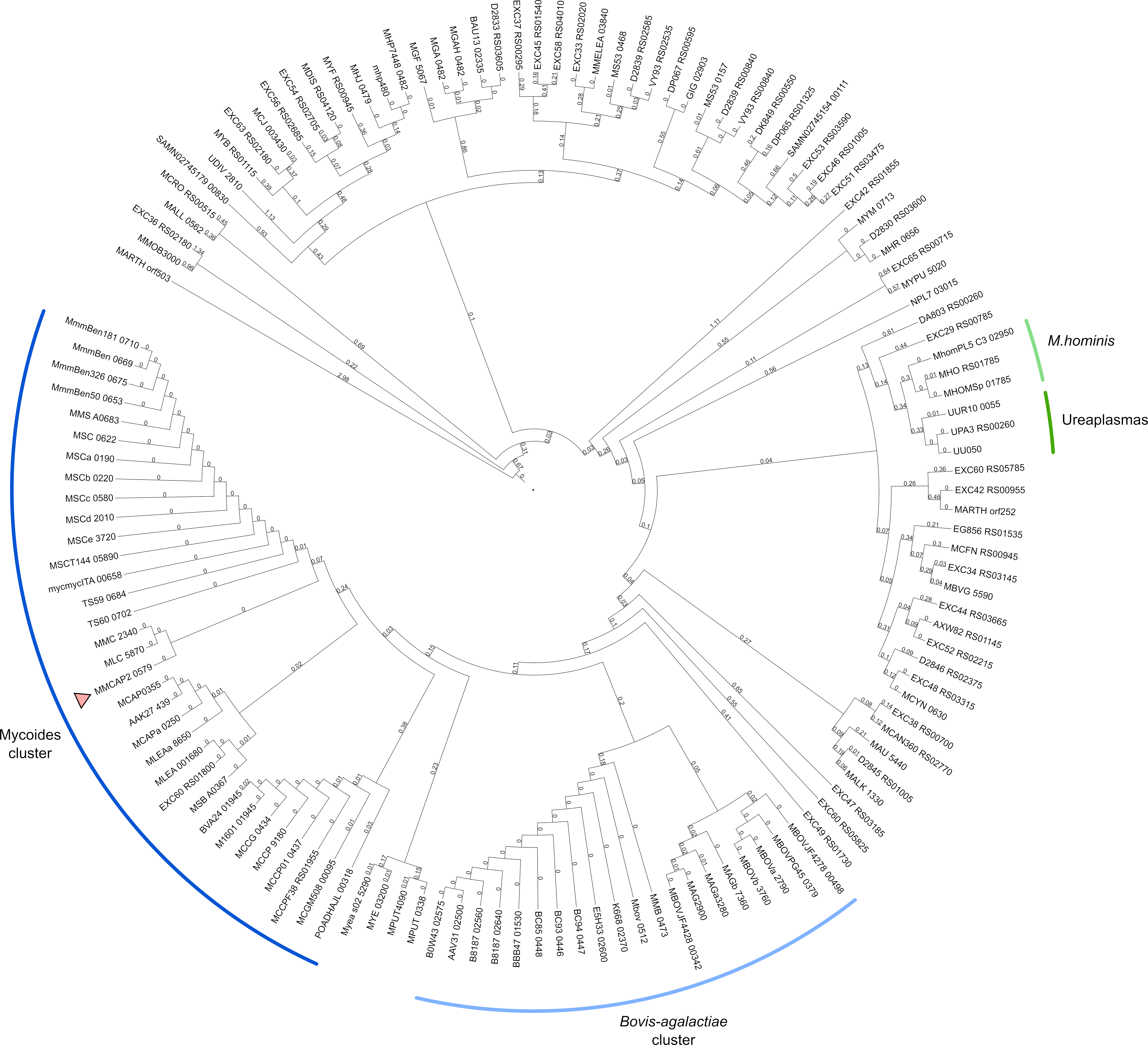

Supplement: S4 Fig — Phylogenetic analysis of the predicted homologs of MMCAP2_0579 was performed using the Phylogeny tool (https://www.phylogeny.fr/) in “One click” mode. The output phylogenic tree was visualized using iTol (https://itol.embl.de/) as a circular tree. The position of MMCAP2_0579 in the tree is marked by the coloured arrowhead. Mycoides cluster and the Bovis-agalactiae cluster are highlighted in blue, showing their phylogenetic proximity, in accordance with a horizontal gene transfer scenario, and similarly for the Hominis and Ureaplasma clusters that are highlighted in green. (TIF) [file ppat.1014043.s004.tif]

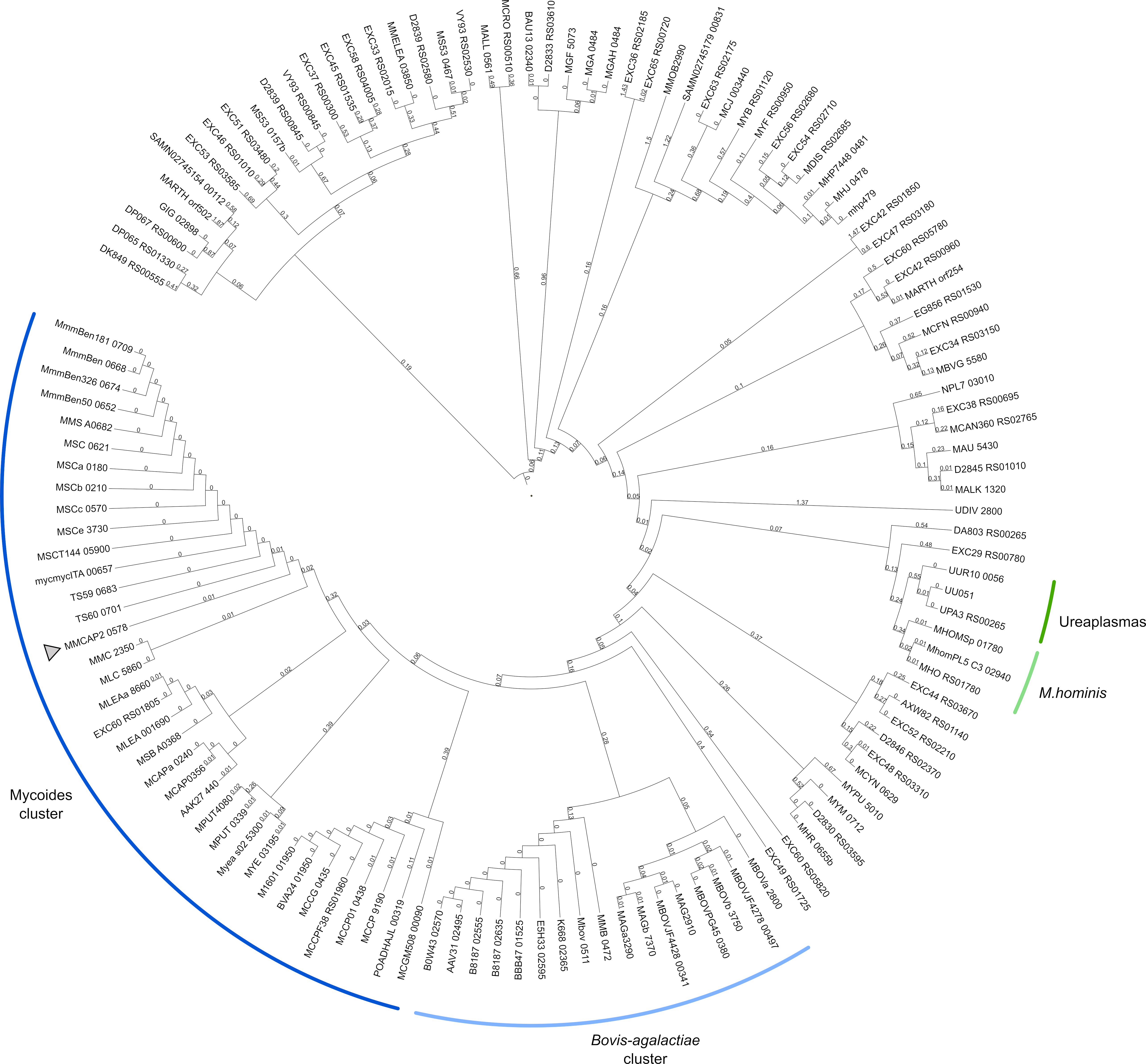

Supplement: S5 Fig — Phylogenetic analysis of the predicted homologs of MMCAP2_0578 was performed using the Phylogeny tool (https://www.phylogeny.fr/) in “One click” mode. The output phylogenic tree was visualized using iTol (https://itol.embl.de/) as a circular tree. The position of MMCAP2_0578 in the tree is marked by the coloured arrowhead. Mycoides cluster and the Bovis-agalactiae cluster are highlighted in blue, showing their phylogenetic proximity, in accordance with a horizontal gene transfer scenario, and similarly for the Hominis and Ureaplasma clusters that are highlighted in green. (TIF) [file ppat.1014043.s005.tif]

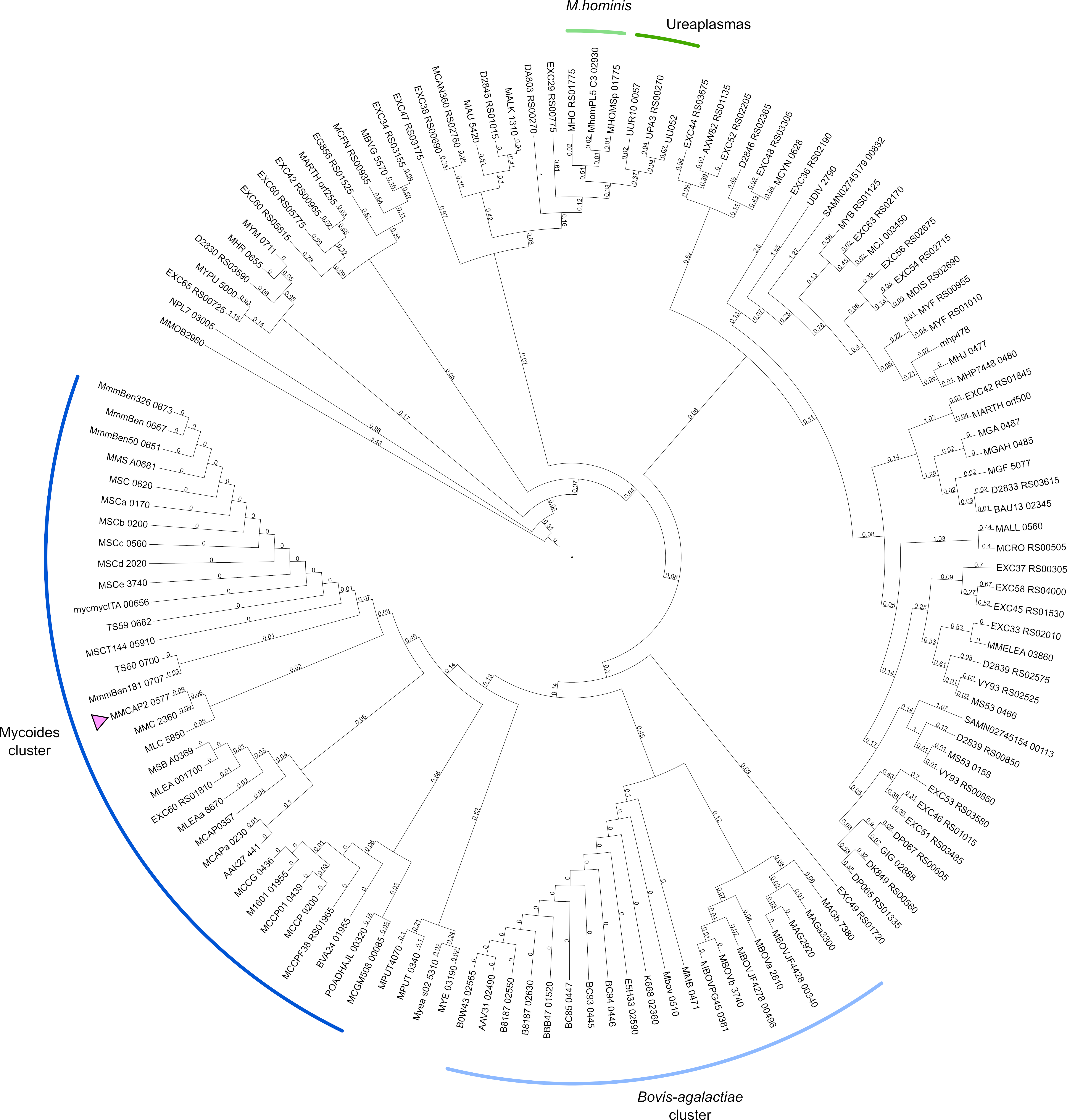

Supplement: S6 Fig — Phylogenetic analysis of the predicted homologs of MMCAP2_0577 was performed using the Phylogeny tool (https://www.phylogeny.fr/) in “One click” mode. The output phylogenic tree was visualized using iTol (https://itol.embl.de/) as a circular tree. The position of MMCAP2_0577 in the tree is marked by the coloured arrowhead. Mycoides cluster and the Bovis-agalactiae cluster are highlighted in blue, showing their phylogenetic proximity, in accordance with a horizontal gene transfer scenario, and similarly for the Hominis and Ureaplasma clusters that are highlighted in green. (TIF) [file ppat.1014043.s006.tif]

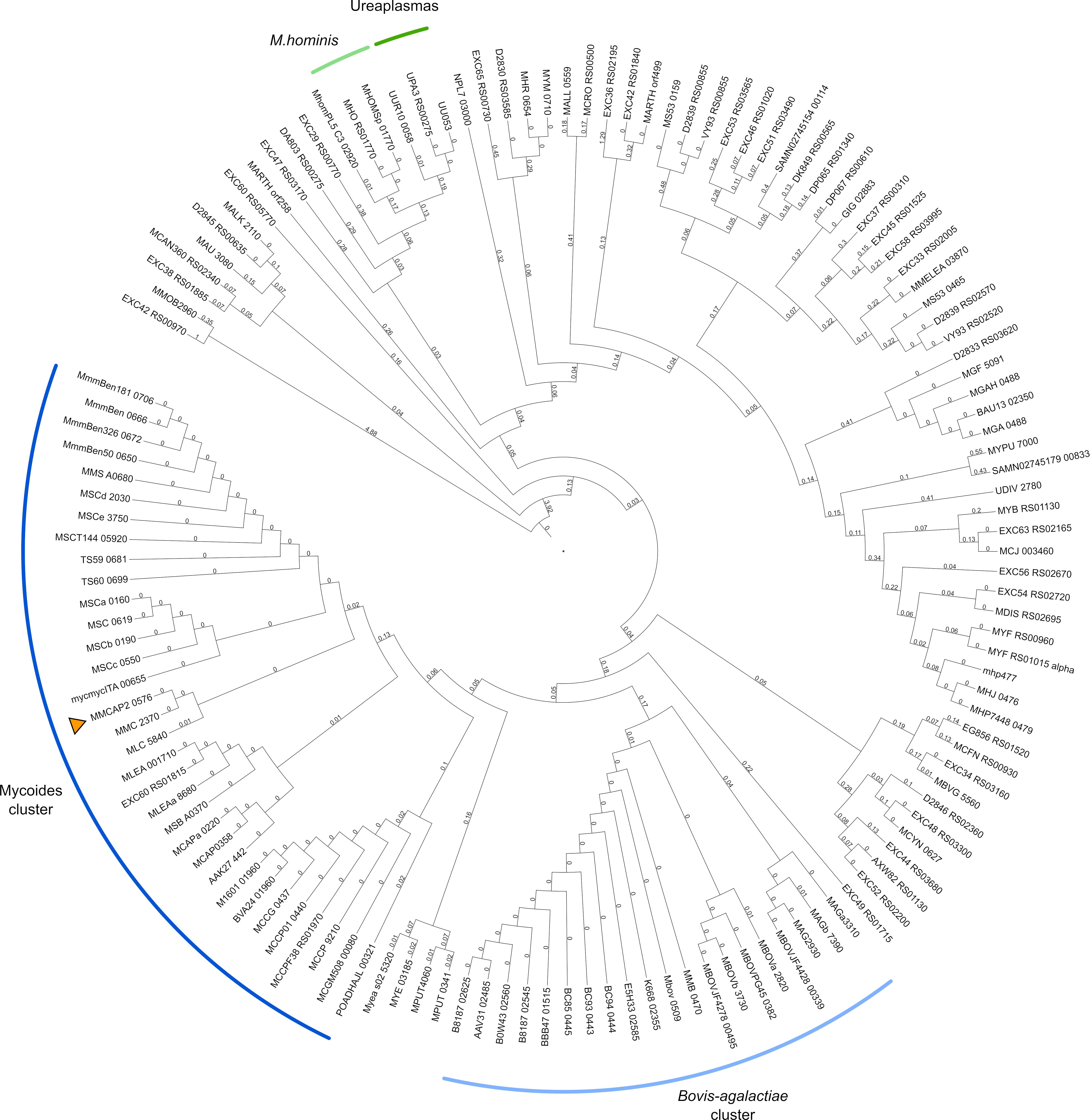

Supplement: S7 Fig — Phylogenetic analysis of the predicted homologs of MMCAP2_0576 was performed using the Phylogeny tool (https://www.phylogeny.fr/) in “One click” mode. The output phylogenic tree was visualized using iTol (https://itol.embl.de/) as a circular tree. The position of MMCAP2_0576 in the tree is marked by the coloured arrowhead. Mycoides cluster and the Bovis-agalactiae cluster are highlighted in blue, showing their phylogenetic proximity, in accordance with a horizontal gene transfer scenario, and similarly for the Hominis and Ureaplasma clusters that are highlighted in green. (TIF) [file ppat.1014043.s007.tif]

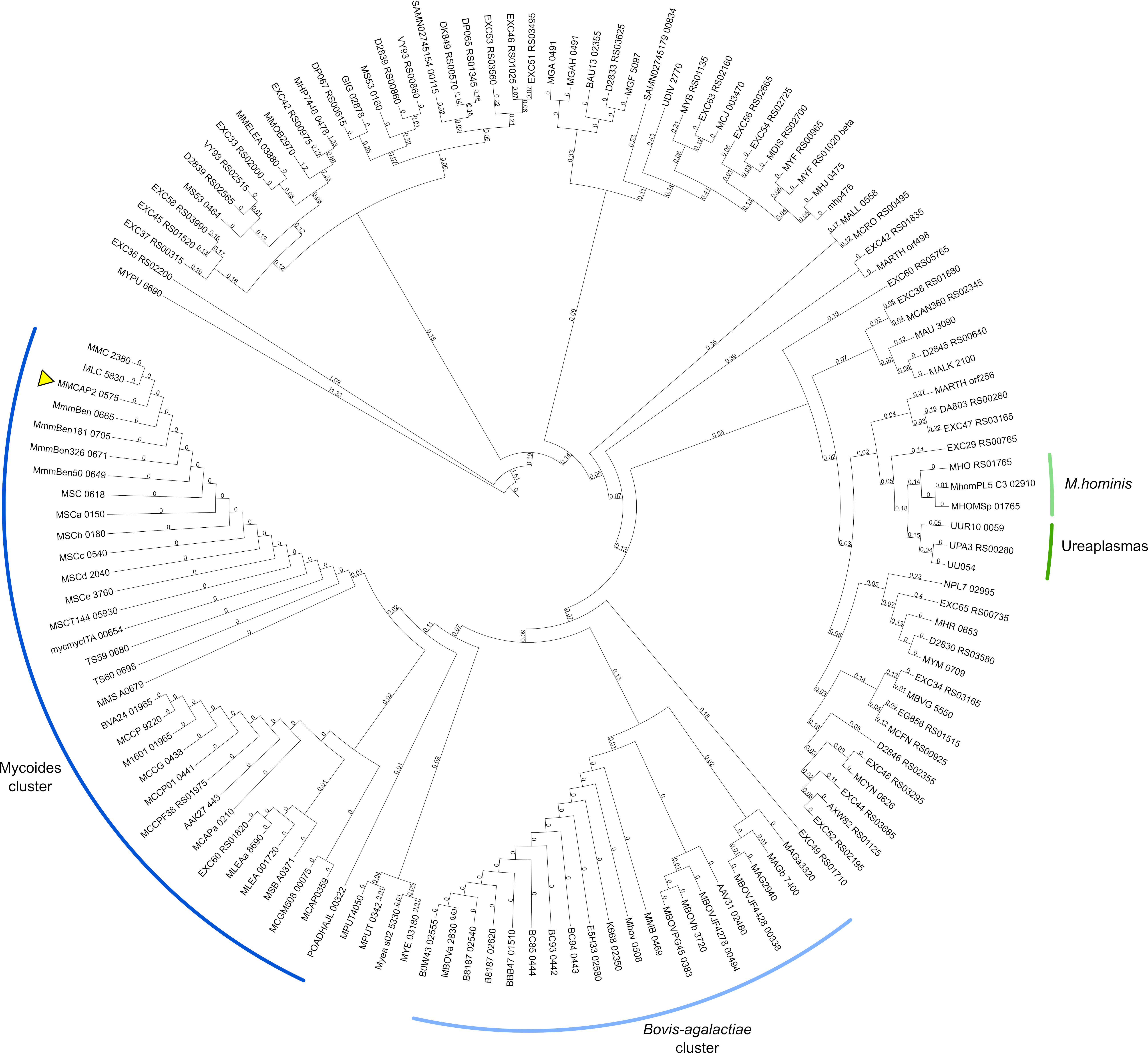

Supplement: S8 Fig — Phylogenetic analysis of the predicted homologs of MMCAP2_0575 was performed using the Phylogeny tool (https://www.phylogeny.fr/) in “One click” mode. The output phylogenic tree was visualized using iTol (https://itol.embl.de/) as a circular tree. The position of MMCAP2_0575 in the tree is marked by the coloured arrowhead. Mycoides cluster and the Bovis-agalactiae cluster are highlighted in blue, showing their phylogenetic proximity, in accordance with a horizontal gene transfer scenario, and similarly for the Hominis and Ureaplasma clusters that are highlighted in green. (TIF) [file ppat.1014043.s008.tif]

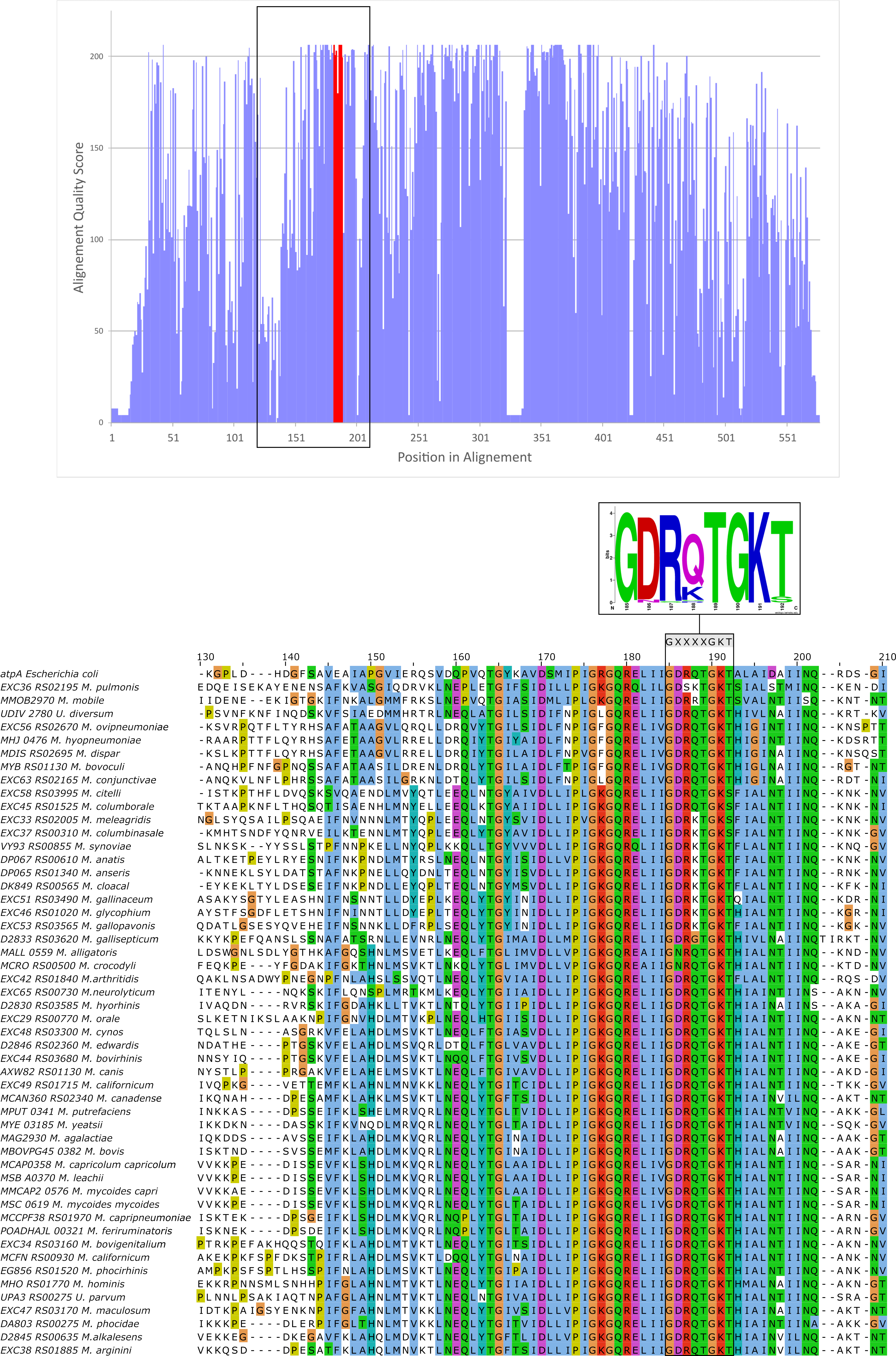

Supplement: S9 Fig — The amino-acid sequences of 51 representative α-like homologs were extracted from S2 Table, as well as the sequence of the protein AtpA from E. coli, and aligned using ClustalW. The alignment quality scores by residue are plotted in purple, and the Walker A motif is highlighted in red (top). The region of the alignment in the black box is detailed below (bottom). The alignment file was opened using JalView (https://www.jalview.org/), and coloured according to the clustal palette. The Walker A consensus motif GXXXGKT is noted in grey, aligned with the predicted Walker A motif in the α and α-like subunits. The sequence logo of the Walker A motif was generated using Weblogo (https://weblogo.berkeley.edu). (TIF) [file ppat.1014043.s009.tif]

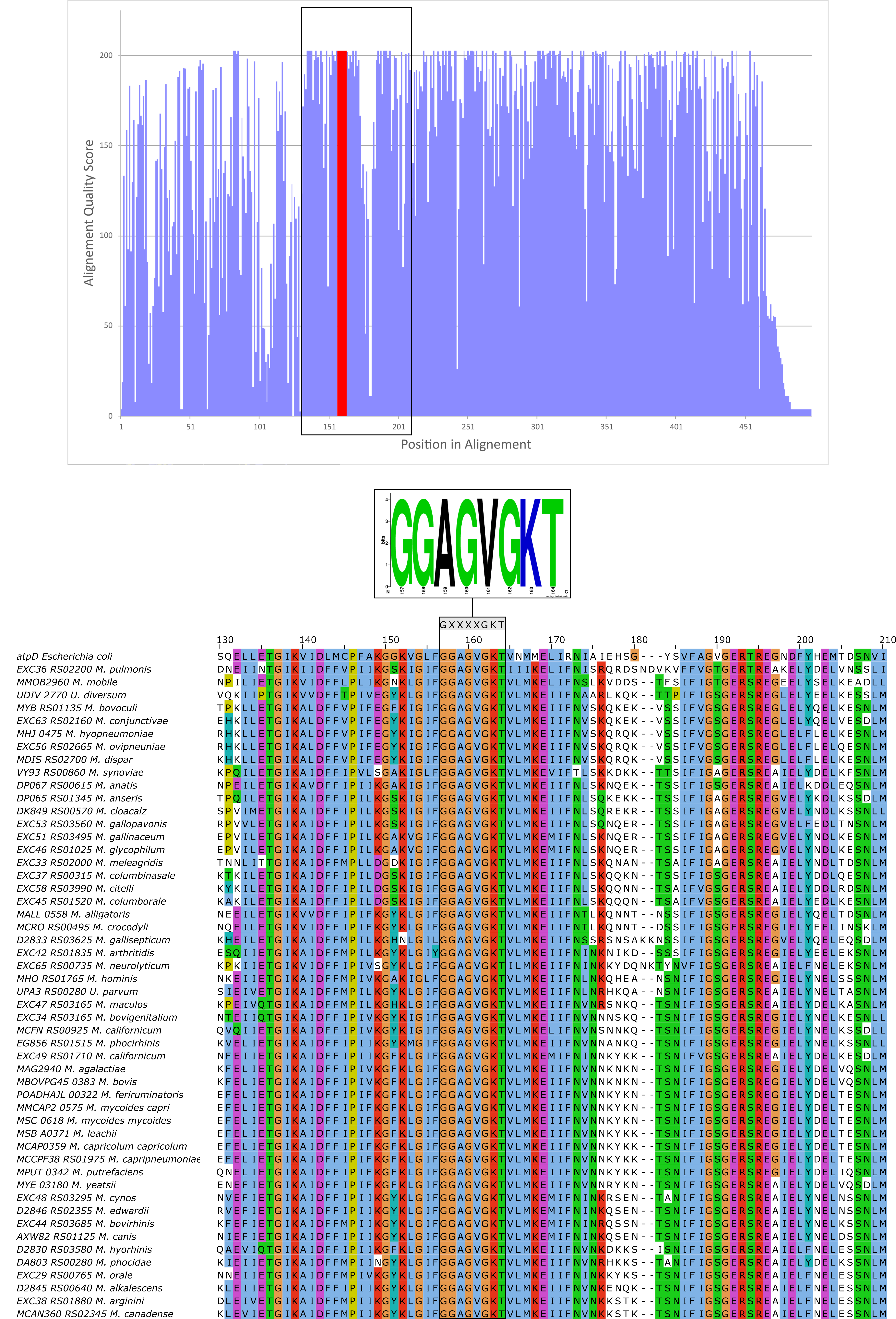

Supplement: S10 Fig — The amino-acid sequences of 51 representative β-like homologs were extracted from S2 Table as well as the sequence of the protein AtpD from E. coli, and aligned using ClustalW. The alignment quality scores by residue are plotted in purple, and the Walker A motif is highlighted in red (top). The region of the alignment in the black box is detailed below (bottom). The alignment file was opened using JalView (https://www.jalview.org/), and coloured according to the clustal palette. The Walker A consensus motif GXXXGKT is noted in grey, aligned with the predicted Walker A motif in the β and β-like sub-units. The sequence logo of the Walker A motif was generated using Weblogo (https://weblogo.berkeley.edu). (TIF) [file ppat.1014043.s010.tif]

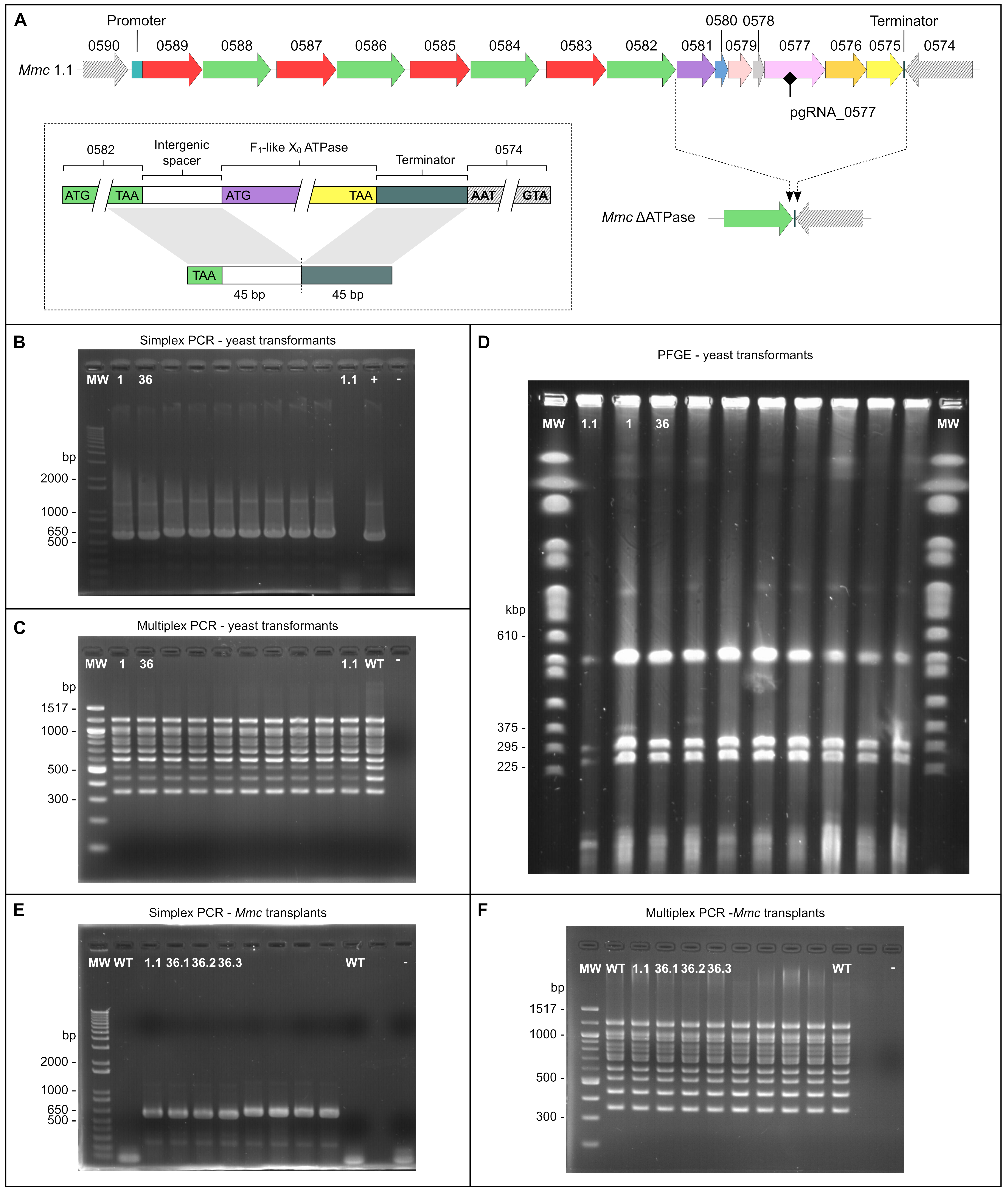

Supplement: S11 Fig — A) The locus encoding the MIB-MIP-F1-like X0 ATPase in Mmc 1.1 is presented using the same pattern as in Fig 1 (top). The position of the sequence targeted by the guide RNA pgRNA_0577 is denoted by a black diamond. The final locus in the mutant Mmc ΔATPase is also displayed (bottom). A detailed view of the recombination arms used to perform the editing in-yeast, following CRISPR-Cas9 stimulated Homologous Recombination is shown (inset). A recombination patch comprised of 2x45 bp homologous arms was generated from two oligonucleotides. The left arm of the patch corresponds to the TAA stop codon of MMCAP2_0582 and the subsequent intergenic spacer. The right arm of the patch corresponds to the terminator of the putative MIB-MIP-F1-like X0 ATPase operon. The double recombination is shown by grey parallelograms. B) Simplex PCR screening of the yeast transformants. The properly edited locus should yield a 555 bp amplicon. “1.1”: Mmc 1.1 gDNA template; “+”: positive control; “-”: no DNA control. C) Multiplex PCR screening of the yeast transformants. The complete genome of Mmc mutants should yield the same 11 amplicons as in the positive control. “1.1”: Mmc 1.1 gDNA template; “WT”: Mmc GM12 gDNA template; “-”: no DNA control. D) PFGE analysis of the bacterial chromosome carried in yeast after restriction by XhoI. The complete genome of Mmc mutants should yield the same 3 large fragments (590, 269 and 226 kpb) as the positive control. “1.1”: Mmc 1.1 gDNA. E) Simplex PCR screening of the bacterial transplants. The properly edited locus should yield a 555 bp amplicon. The lane 1.1 corresponds to the transplant 1 obtained from the yeast clone 1. The lanes 36.1, 36.2 and 36.3 correspond to the 3 transplants obtained from the yeast clone 36. “WT”: Mmc GM12 gDNA template; “+”: positive control; “-”: no DNA control. F) Multiplex PCR screening of the bacterial transplants. The complete genome of Mmc mutants should yield the same 11 amplicons as in the positive control. “WT”: Mmc GM1 [file ppat.1014043.s011.tif]

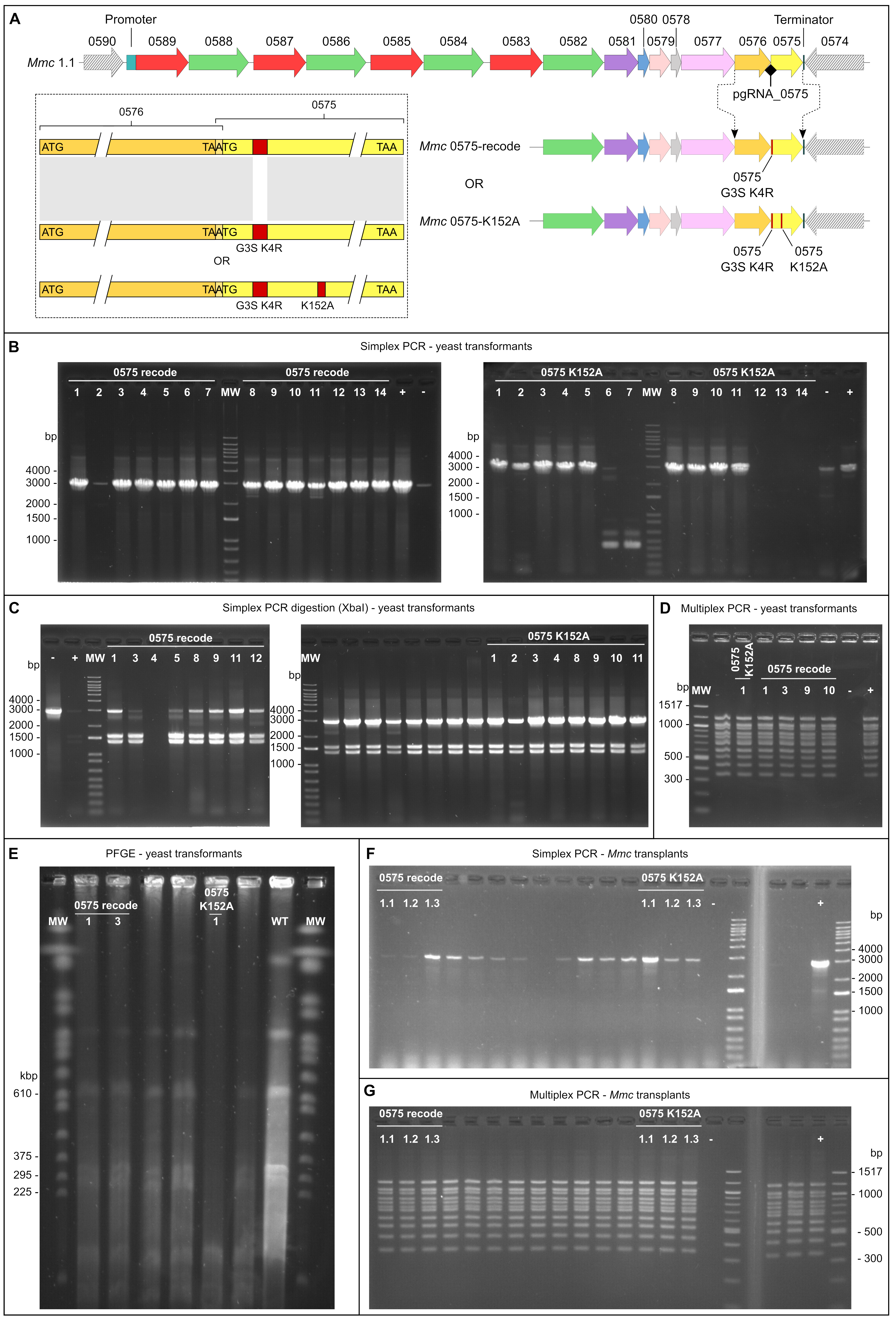

Supplement: S12 Fig — A) The locus encoding the MIB-MIP-F1-like X0 ATPase in Mmc 1.1 is presented using the same pattern as in Fig 1 (top). The position of the sequence targeted by the guide RNA pgRNA_0575 is denoted by a black diamond. The edited locus in the mutants Mmc 0575-recode or Mmc 0575-K152A is also displayed (middle and bottom). A detailed view of the recombination arms used to perform the editing in-yeast, following CRISPR-Cas9 stimulated Homologous Recombination is shown (inset). A recombination patch comprised of the complete and edited locus MMCAP2_0576-MMCAP2_0576 was generated by PCR. The left arm of the patch corresponds to the complete wild-type locus MMCAP2_0576. The right arm of the patch corresponds to theedited locus MMCAP2_0575-recode or MMCAP2_0575-K152A. The double recombination is shown by grey parallelograms. B) Simplex PCR screening of the yeast transformants. The properly edited locus should yield a 2927 bp amplicon. “+”: positive control; “-”: no DNA control. C) XbaI restriction screening of the Simplex PCR amplicons. The properly edited locus should yield two bands at 1554 bp and 1373 bp. “+”: XbaI restriction positive control; “-”: XbaI restriction negative control. D) Multiplex PCR screening of the yeast transformants. The complete genome of Mmc mutants should yield the same 11 amplicons as in the positive control. “1.1”: Mmc 1.1 gDNA template; “-”: no DNA control. E) PFGE analysis of the bacterial chromosome carried in yeast after restriction by XhoI. The complete genome of Mmc mutants should yield the same 3 large fragments (590, 269 and 226 kpb) as the positive control. “1.1”: Mmc 1.1 gDNA. F) Simplex PCR screening of the bacterial transplants. The properly edited locus should yield a 2927 bp amplicon. The lane 1.1, 1.2 and 1.3 corresponds to the transplants 1, 2 and 3 obtained from the yeast clone 1. “+”: positive control; “-”: no DNA control. G) Multiplex PCR screening of the bacterial transplants. The complete genome of Mmc mutants should yield the [file ppat.1014043.s012.tif]

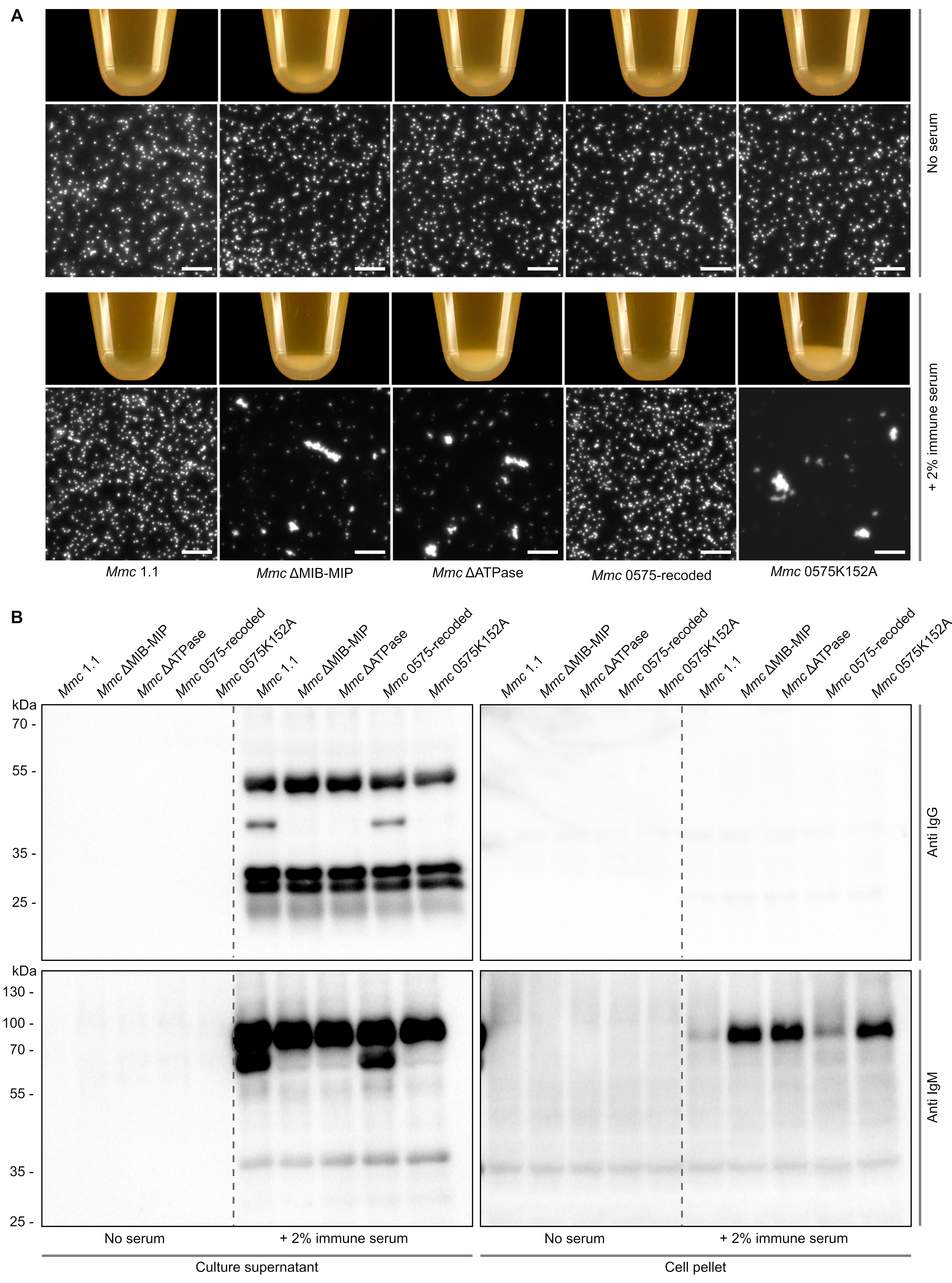

Supplement: S13 Fig — A) Agglutination of cells by immune goat serum. The cells were grown in axenic conditions, in absence (top) or presence (bottom) of 2% of serum from a goat experimentally immunized, collected 12 days post-inoculation. The bottom of the micro-centrifuge tubes was photographed (top). After resuspension of the flocculates by inversion of the tubes, a sample was mounted between a glass slide and a coverslip and was imaged on a dark-field microscope (bottom - scale bar: 10 µm. B) Western blot analysis of the immunoglobulin Heavy Chain cleavage by the MIB-MIP system. Samples corresponding to either culture supernatant or cell pellet derived from the agglutination assays were separated by SDS-PAGE, then analyzed by Western Blotting using primary antibodies targeting either the goat IgG Fc or the goat IgM Heavy Chain. Intact Heavy Chain and MIB-MIP cleaved Heavy Chain are highlighted by a gray and white arrowhead, respectively. (TIF) [file ppat.1014043.s013.tif]

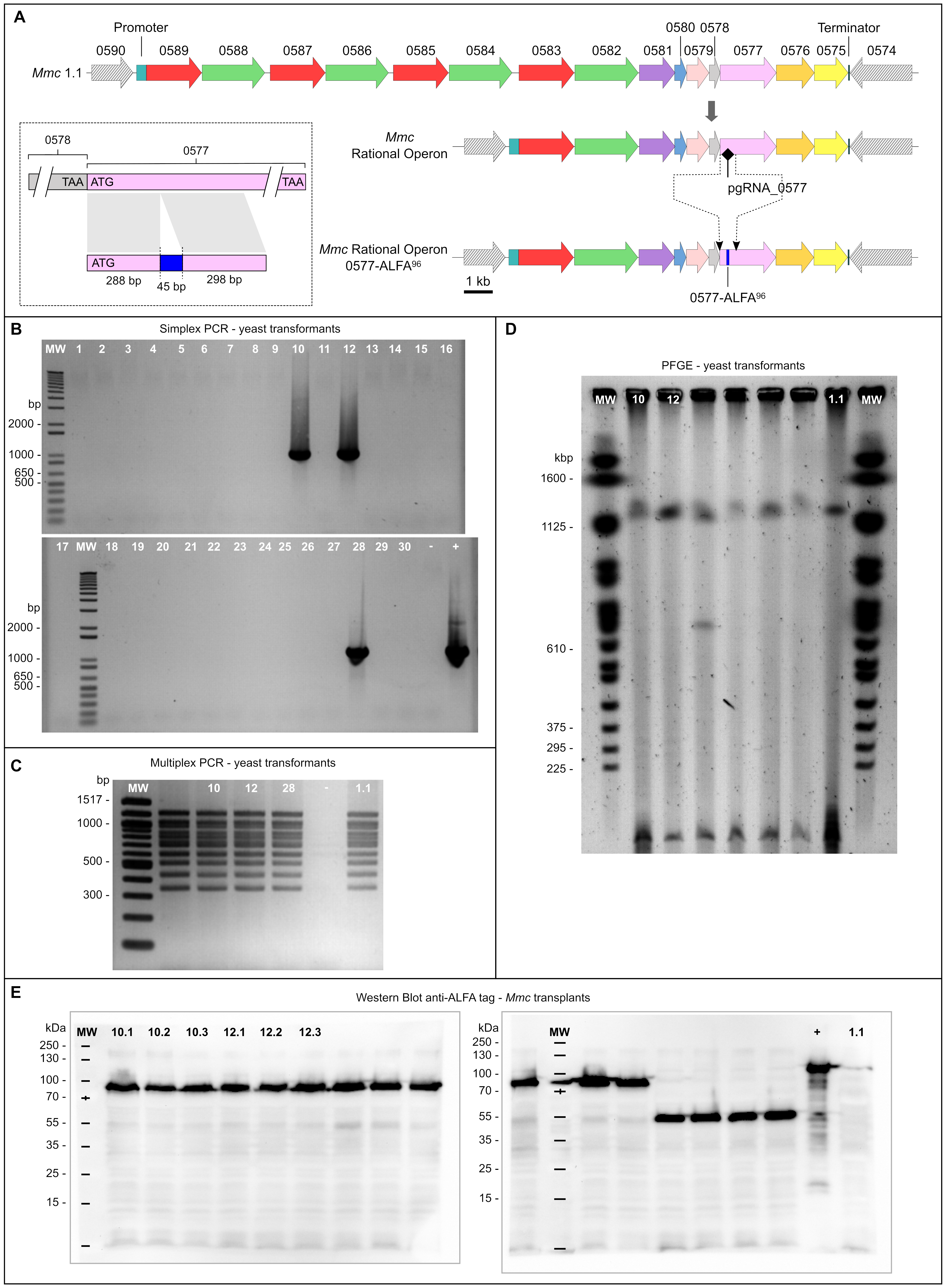

Supplement: S14 Fig — A) The locus encoding the MIB-MIP-F1-like X0 ATPase in Mmc 1.1 is presented using the same pattern as in Fig 1 (top). The locus in the mutant Mmc Rational Operon, generated in a previous study, is also shown (middle). The position of the sequence targeted by the guide RNA pgRNA_0577 is denoted by a black diamond. The final locus in the mutant Mmc Rational Operon 0577-ALFA96 is also displayed (bottom). A detailed view of the recombination arms used to perform the edition in-yeast, following CRISPR-Cas9 stimulated Homologous Recombination is shown (inset). A recombination patch comprised of two ~ 290 bp homologous arms was generated by PCR. The left arm of the patch corresponds to 288 first coding bases of MMCAP2_0577. The right arm of the patch corresponds to the next 298 bp of MMCAP2_0577. The two arms are separated by 45 bp encoding the ALFA tag (13x3 bp) flanked on each side by two serine encoding codons (2x3 bp). The double recombination is shown by grey parallelograms. B) Simplex PCR screening of the yeast transformants. The properly edited locus should yield a 1012 bp amplicon. “+”: positive control; “-”: no DNA control. C) Multiplex PCR screening of the yeast transformants. The complete genome of Mmc mutants should yield the same 11 amplicons as in the positive control. “1.1”: Mmc 1.1 gDNA template; “-”: no DNA control. D) PFGE analysis of the bacterial chromosome carried in yeast. The complete genome of Mmc mutants should yield the same 1 large fragment (994 kpb) as the positive control. “1.1”: Mmc 1.1 gDNA. E) Anti-ALFA tag Western blot screening of the bacterial transplants. For each analyzed transplant, whole cell extracts were generated and separated by SDS-PAGE. Anti-ALFA Western blotting was then performed. The Mmc mutants should display a single band at ~85 kDa. The lanes 10.1, 10.2, 10.3 and 12.1,12.2, 12.3 corresponds to the 3 transplants obtained from the yeast clone 10 and 12, respectively. “+” recombinant ALFA-tagged protein; “1.1”: Mmc 1.1 whole [file ppat.1014043.s014.tif]

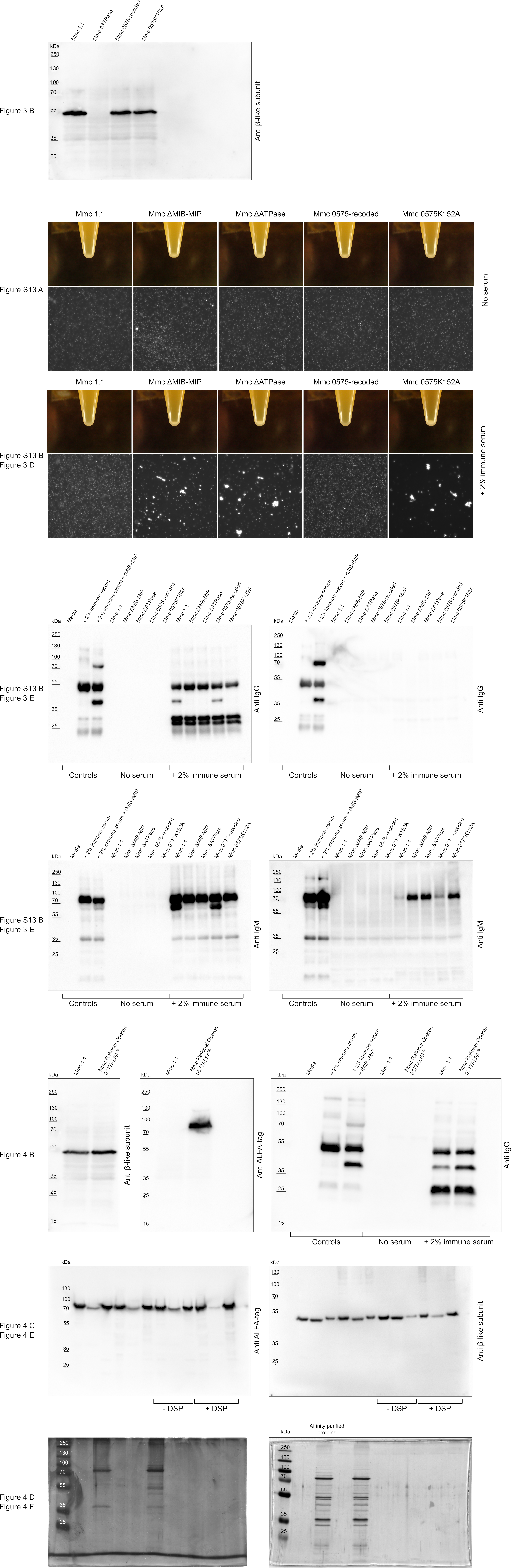

Supplement: S15 Fig — The Figs of this publication contain images that have been cropped and adjusted for contrast or levels. Cropping has been performed to improve legibility or to remove internal control lanes. Adjustments have been applied homogeneously to all images. Image processing does not modify the interpretation of the images. Original uncropped and unadjusted images are provided for reference. (TIF) [file ppat.1014043.s015.tif]
