# Supplementary material for: An atypical F-type ATPase is necessary for the function of the antibody cleavage system MIB-MIP in mycoplasmas
Source: PLoS Pathog. 2026 Mar 11;22(3):e1014043. doi: 10.1371/journal.ppat.1014043 (PMC12994834; doi:10.1371/journal.ppat.1014043)
Supplement: Attachment — Submitted filename: Response to Reviewers.docx [file ppat.1014043.s023.docx]

Yonathan Arfi

UMR 1332 BFP – INRAE – Univ. Bordeaux

Bat. A4 – IBVM, Equipe Mollicutes

71 avenue Edouard Bourlaux

33140 Villenave d’Ornon, France

Villenave d’Ornon, December 5^th^ 2025

To:	the PLOS Pathogens Editorial Team

the Reviewers of manuscript PPATHOGENS-D-25-03106

Subject: Response to Reviewers for manuscript PPATHOGENS-D-25-03106

Dear PLOS Pathogens Editorial Team, dear Reviewers,

My co-authors and I would like to thank you for your very positive evaluation of our manuscript “An atypical F-type ATPase is necessary for the function of the antibody cleavage system MIB-MIP in mycoplasmas”.

You will find below a detailed response to your comments and questions, that we hope will be satisfactory. Please note that our responses will follow the order outlined in the Decision Letter (Part I: Summary; Part II: Major Issues; Part III: Minor Issues). We have also amended parts of our manuscript in order to reflect some of your comments. These edits are listed in the responses to comments, and highlighted in the provided marked-up copy of the revised manuscript.

We also want to specifically point out the following change in the manuscript: Panel D of Figure 4 has been replaced (as well as the corresponding uncropped image on Figure S15). Indeed, during the production of the revised manuscript, we have become aware of a mistake during the selection of the corresponding image. This change has no effect on the manuscript claims, interpretations and conclusions. The comparison of the initial and revised images, as well as an explanation for the misattribution is given at the end of the detailed response to comments and questions.

We look forward to reading your feedback regarding the suitability of this revised manuscript for publication in PLOS Pathogens.

Yours faithfully,

Yonathan Arfi, PhD

**Part I - Summary**

**Reviewer #1:**

1) *The authors posit from phylogenetic evidence that horizontal gene transfer is the likely to have occurred several times during the evolution of the Mollicutes. It there evidence for this e.g. G + C content disparity? Is it not possible that the clusters evolved early in the last universal common ancestor and is undergoing decay in specific lineages?*

The notion that the MIB-MIP-ATPase system has probably been exchanged through HGT is indeed based on phylogenetic analysis. It is based on the observed close proximity between the systems found in the genomes of phylogenetically distant species such as the *Mycoplasma bovis*-*Mycoplasma agalactiae* group and the Mycoides cluster; and *Mycoplasma hominis* and *Ureaplasma* spp. Interestingly, these possible exchanges are found across mycoplasma species that share the same host species (ruminants and human, respectively). In this manuscript, we provide phylogenetic analysis only for the individual components of the ATPase clusters, but the same pattern can be observed for MIB and MIP (see our previous publications: doi.org/10.1073/pnas.1600546113 and doi.org/10.1126/sciadv.abf2403).

One possibility to explain this proximity would be HGT of the system, a hypothesis that is bolstered by the numerous publications describing the phenomenon in mycoplasmas (see doi.org/10.1128/mbio.01958-14; doi.org/10.21775/cimb.029.003; doi.org/10.3389/fmicb.2019.02441; doi.org/10.1371/journal.pgen.1007910). The Reviewers proposition of an emergence of the cluster in a last common ancestor coupled to decay is also valid, although in our view less probable.

The emergence of the MIB-MIP-ATPase system in Mollicutes is currently a nebulous question, and we do not have a clear model to explain the current distribution of the system, as well as it’s almost complete loss in some species such as *Mycoplasma genitalium* and *Mycoplasma pneumoniae*. Based on our current understanding, we propose that the system could have emerged early in the Mollicute’s history, and then been subject to specialization to match the host species. In some cases, the system was lost when selection pressure to maintain it was lifted (in *M. fermentas* for instance; or in plant or arthropod pathogenic Mollicutes for instance). The specific evolution of Type 2 and Type 3 ATPases is also mysterious and we only have hypotheses at this point.

2) *Line 228. The authors dispel the detection of many (but not all) mutations because of their presence in intergenic regions within mobile genetic elements. Was there evidence growth rate compliance among all the mutants and wild type- yes you tested those in the 23SrRNA gene but in other cases, like “amino acid substitutions in proteins of unknown function”. The way this is written implies that genes of unknown function are insignificant. There are likely a myriad of important gene functions yet to be determined.*

We agree with the Reviewer’s comment that the fact that a mutation occurred in an intergenic region or a region of unknown function does not mean that it is biologically insignificant. We do agree that some indirect/knock-on effects might be occurring, which is why we elected to confirm that there was no major impact using targeted (western blot) and untargeted (proteomics) survey of expression in our mutants. The message we wanted to convey with this section was that although some mutations had occurred in our mutants (in particular *Mmc* ΔATPase), none could be directly linked to the modified phenotype we then observed during our experiments.

To reflect this, we have modified Line 228, which now reads “None of these mutations appeared to be directly correlated to the phenotypes observed in our subsequent experiments,”.

3) *Further to this- the section commencing on line 236 indicates that the authors could not find gross differences in proteome content as assessed by western blotting and LC-MS/MS. However these cannot account for any potential* *posttranslational modification differences*

We agree with the Reviewer’s comment that posttranslational modifications are not taken into account in our experiments. However, we do not believe that any part of the MIB-MIP-ATPase system is prone to these modifications. Furthermore, we do not believe that any of the observed SNPs and INDELs could be directly correlated to variations in these modifications (although, as stated above, we agree that this was not measured).

To reflect the Reviewer’s concern, we have added a sentence (now at Line 253) that reads “ *[…] no significant difference to the control could be observed in the abundances of the 13 proteins encoded in the locus.* We nonetheless note that our experiments cannot rule out the existence of differences in posttranslational modifications, although these should not affect the MIB-MIP-ATPase components.”.

**Reviewer #2:**

There was no specific comment to address in this section.

**Reviewer #3:**

There was no specific comment to address in this section.

**Part II – Major Issues**

**Reviewer #1:**

There was no specific comment to address in this section.

**Reviewer #2:**

1) *L103) Description and structure prediction of the putative F1-like X0 ATPase of Mmc: I recommend to include a typical predicted structure like Fig. S1B or D in Fig. 1.*

We strongly disagree with the Reviewer’s recommendation that the predicted structure shown in Fig S1B or D should be in the Figure 1. Indeed, these predicted structures are only that: predictions, some of which have limited confidence scores (as mentioned by Reviewer #3). We have internally debated whether or not to include these predictions of multi-protein complexes, and eventually opted to only show them in the SI, in order not to give them more attention than necessary. Visualizations of these “structures” could be taken at face value by some readers, and convey a potentially incorrect model. While we rely on this model for our discussion, and for the diagram in Figure 5, we believe that a schematic does not convey the same impact as a 3D structure. We will hopefully one day have an experimentally solved 3D structure of the ATPase, which will then be in a main text Figure.

2) *L321) We eventually succeeded by adding the sequence of the ALFA-tag, a 13 amino-acids epitope tag for which a cognate Nanobody is available [39], after the residue S96 (Fig 4A). The authors should show evidences of “membrane” fraction, based on mass spec, electron microscopy, optical microscopy and so on.*

We apologize to the Reviewer, but we do not understand the meaning of their comment. The presence of the ALFA-tagged protein in the membrane fraction is presented in Figures 4C and 4E. In these panels, we show an anti-ALFA western blot performed on either the soluble or the isolated membrane fraction. Could the Reviewer clarify their comment so that we may better respond?

3) *L422) assay. As a result, cleaved Heavy Chain might be present at the cell surface, but our assay might not be sensitive or specific enough to detect them. How about possibility to track antibodies with an intense fluorescent dye as a future work?*

Yes, using fluorescence labelled antibodies to perform the *in cellulo* cleavage assay, followed by performing super-resolution microscopy (dSTORM), was studied. However, this would result in seeing an intense fluorescence signal on both the mutants and the WT strains, as antibodies would bind to their cognate antigens no matter what. The proteolytic cleavage would not be visible using this strategy. We are currently trying to develop a method to first enrich the membrane fraction, then perform antibody isolation from this fraction, followed by a step of affinity depletion of functional antibodies, in order to have a higher signal from the cleaved antibodies fraction. However, we believe that this is beyond the scope of this manuscript.

**Reviewer #3:**

There was no specific comment to address in this section.

**Part III – Minor Issues**

**Reviewer #1:**

1) Grammatical error at Line 252 was corrected

2) *Line 327. Please explain “a detergent selected after an initial screening (data not shown). What type of screening are you meaning- please explain the statement.*

Several detergents can be used for membrane proteins solubilization. The use of DDM was not initially self-evident (it is one option among many), and a typically initial experiment is to compare different detergent to assess which one is the most suitable for each individual protein. This type of “detergent screening” is standard practice in membrane protein purification, and we performed ours using two kits from Cube Biotech (Detergent Screening Set Classic and Detergent Screening Set 2) which each contains the detergents OG, LDAO, DM, DDM and OTG; and NG, NM, UDM, TDM and CHAPS, respectively.

To clarify this point, the text now reads: “[…] by addition of n-dodecyl-β-D-maltoside (DDM), a detergent that was selected after an initial screening of several detergents (Cube Biotech, Detergent Screening Set Classic and Detergent Screening Set Classic 2, which respectively contain the detergents OG, LDAO, DM, DDM and OTG , and NG, NM, UDM, TDM and CHAPS; data not shown).

3) *Line 463. The proteomic determination of the F1ATPase components were first identified in M. hyopneumoniae in the paper by Tacchi et al., 2016 (Open Biol. 2016 Feb;6(2):150210. doi: 10.1098/rsob.150210.-supplementary data), preceding Reference 65.*

The goal of this section was not to specifically cite the first occurrence of each publication in which proteomic analysis was performed, but merely to illustrate that the genomics analysis we performed is supported by actual expression data in some relevant species. We have updated reference 65 to match the reference suggested by the Reviewer.

**Reviewer #2:**

1) *L62) by twisting the variable domains of the Heavy and Light Chains of immunoglobulins. Explain the information to suggest twisting.*

The “twisting” mentioned in the sentence is fully described in the cited reference 22 (doi.org/10.1126/sciadv.abf2403). Binding of MIB to the IgG light chain leads to a shift in the relative alignment of the VH and VL domains, twisting them out of position (see Figure 3 of reference 22).

2) *L90) and the rare Type 2 which corresponds to the molecular motor involved in energizing the motility in Mycoplasma mobile [29,30]. The ATPase unit of Type 2 ATPase is designated also as “G1-ATPase” in reference [43]. Refer to this name at an early position.*

Indeed, Type 2 ATPase have been recently termed G1-ATPase (“G” for Gliding) by the group led by Reviewer 2. We were aware of this nomenclature, but given its recency, we were unsure of whether to use it or not. In order to match the Reviewer’s comment, we have edited line 90, which now reads “Two other types of F-ATPase are found in mycoplasmas: the ubiquitous Type 1 which corresponds to the essential F_1_F_0_ ATP synthase; and the rare Type 2 which corresponds to the G_1_-ATPase, to the molecular motor involved in energizing the gliding motility in *Mycoplasma mobile* [29,30].”

We have also edited the manuscript in multiple places: every time the text read “Type 2 F-ATPase” we replaced it by “Type 2 G_1_-ATPase”.

3) *L129) and many) F1-like complex. Check subscript, italic, capitarization, especially for “References”.*

We have checked the manuscript in order to homogenize the writing as “F_1_-like X_0_”.

4) L191) an extremely strong genetic linkage. This sounds too strong. “strong genetic linkage” may be enough.

Sentence has been edited and now reads “a strong genetic linkage”.

5) *L271) exactly the same agglutinated phenotype. This sounds too strong. The pellet appearances are not the same. “very similar agglutination phenotype” may be enough.*

Sentence has been edited and now reads “very similar agglutination phenotype”.

6) *L293) 0575-recode. Does this mean a 0575 mutant complemented by an original 0575? Clarify it.*

No, the nomenclature “0575-recode” corresponds to a Mmc mutant which is “quasi-WT”, but had to be edited in order to change a PAM motif, and as a result has mutations G3S and K4R in MMCAP2_0575. The nomenclature is fully explained in the preceding text, at lines 211-216, reading “However, synonymous recoding of the PAM was not possible, and it was therefore necessary to perform a non-synonymous recoding which yielded the substitutions G3S and K4R. In order to confirm that these two undesired modifications in the N-terminal part of MMCAP2_0575 had no effect, we have thus generated an independent mutant, termed “Mmc 0575-recode” in which the substitutions G3S and K4R are present, but the lysine 152 is maintained (Fig 3A).”

7) *L302) We eventually succeeded by adding the sequence of the ALFA-tag, a 13 amino-acids epitope tag for which a cognate Nanobody is available [39], after the residue S96 (Fig 4A). Which protein did you choose? Clarify it.*

After re-reading, the corresponding sentence can indeed be mis-interpreted. Therefore, the sentence was amended for clarity and now reads: “after the residue S96 of MMCAP2_0577 (Fig 4A).”.

8) *L405) which in turn pulls on a substrate binding adhesin (the “leg”) (Fig 5A). Is “on” necessary?*

Sentence has been edited and now reads “which in turn pulls a substrate binding adhesin”

**Reviewer #3:**

1) *I did wonder about whether the authors looked into whether binding of antibody to the cognate antigen on Mmc is required for the activation of the MIB-MIP system, or if the system is able to cleave non-specific antibodies.*

Yes, this was indeed studied previously, and the data can be found in a previous publication (doi.org/10.1126/sciadv.abf2403). Briefly: the system appears to only process antibodies that are bound to a cognate antigen “in vivo”, meaning that if one tries to perform an *in cellulo* cleavage assay with “naïve” antibodies (that do not target the cell surface) no cleavage occurs. However, in vitro, using recombinant MIB and MIP, one can cleave any antibody. So, in our model, the MIB-MIP system does not “reach out” to the media to pick and cleave antibodies, but rather “waits” for an antibody to stay/ reside at the cell surface. It acts as a form of “point defense system” that processes antibodies actually “imobilized” on the cell surface.

2) *Could it be that the ATPase is serving more so to localize the MIB-MIP system to the site of antibody bound to the surface of the cell rather than energizing the MIP to cleave? Or is the current thought that MIB just binds whichever antibody is in close proximity to it?*

Well, we think that maybe both… We now know that energy consumption is required for the MIB-MIP system to cycle. But that it not contradictory with the ATPase being the “organizing” center of MIB-MIP. In one of our models, MIB and MIP are arranged around the ATPase membrane domain X0, forming a rosette-like structure (akin to petals on a flower). In this configuration, MIB shields the surrounding antigen. In an alternative model, MIB is “roaming” freely, and “scans” the cell surface to look for bound antibodies, eventually bringing them back to MIP, and eventually the ATPase. We have fluorescence experiments in the pipeline in order to check for possible co-localization of MIB-MIP and ATPase, but working in mycoplasmas is quite challenging…

**Modification of Figure 4D:**

We have replaced the image in the panel of Figure 4D. Below are the images found in the original submission (left), and the replacement image (right).

The original image corresponds to the analysis of the affinity-purified proteins (Nanobody anti-ALFA resin, staining using Coomassie blue) isolated from the non-crosslinked membrane fraction of the strain “*Mmc* Rational Operon 0577-ALFA^96^ 0575-K152A”. This strain combines both the mutations “Rational Operon” (rationalization of the number of MIB and MIP coding sequences), “0577-ALFA^96^” (tagging of MMCAP2_0577 with the ALFA tag to enable affinity purification) and “0575-K152A” (inactivation of the beta-subunit of the type 3 F-ATPase). This strain is not described in the manuscript and was constructed as part of our ongoing efforts to purify the full F_1_like X_0_ ATPase without relying on cross-linking.

The replacement image corresponds to the analysis of the affinity-purified proteins (Nanobody anti-ALFA resin, staining using a combination of Coomassie blue followed by silver staining) isolated from the non-crosslinked membrane fraction of the strain “*Mmc* Rational Operon 0577-ALFA^96^” as indicated in the manuscript.

Both images show the same purification products: the main band at ~85 kDa which corresponds to MMCAP2_0577-ALFA^96^; and a secondary band at ~40 kDa which was then identified as MMCAP2_0581 by mass spectrometry. Given the similarities between the purification outcomes and the strains names, we mistakenly selected an incorrect image when assembling the Figure 4.

We believe that this change of image has no effect on the manuscript claims, interpretation and conclusion. We apologize to the Reviewers and Editorial team for providing an inaccurate Figure 4 panel.
